# Autonomic and muscular responses and recovery to one-hour laboratory mental stress in healthy subjects

**DOI:** 10.1186/1471-2474-8-81

**Published:** 2007-08-14

**Authors:** Kristian B Nilsen, Trond Sand, Lars J Stovner, Rune B Leistad, Rolf H Westgaard

**Affiliations:** 1 Norwegian University of Science and Technology, Faculty of Medicine, Department of Neurosciences, N-7489 Trondheim, Norway; 2 Norwegian University of Science and Technology, Faculty of Social Sciences and Technology Management, Department of Industrial Economics and Technology Management, N-7491, Trondheim, Norway; 3 St. Olavs Hospital Trondheim University Hospital, N-7489 Trondheim, Norway

## Abstract

**Background:**

Stress is a risk factor for musculoskeletal pain. We wanted to explore stress related physiology in healthy subjects in order to gain insight into mechanisms of pain development which may relate to the pathophysiology of musculoskeletal pain disorders.

**Methods:**

Continuous blood pressure, heart rate, finger skin blood flow, respiration, surface electromyography together with perception of pain, fatigue and tension were recorded on 35 healthy women and 9 healthy men before, during a 60 minute period with task-related low-grade mental stress, and in the following 30 minute rest period.

**Results:**

Subjects responded physiologically to the stressful task with an increase in trapezius and frontalis muscle activity, increased blood pressure, respiration frequency and heart rate together with reduced finger skin blood flow. The blood pressure response and the finger skin blood flow response did not recover to baseline values during the 30-minute rest period, whereas respiration frequency, heart rate, and surface electromyography of the trapezius and frontalis muscles recovered to baseline within 10 minutes after the stressful task. Sixty-eight percent responded subjectively with pain development and 64% reported at least 30% increase in pain. Reduced recovery of the blood pressure was weakly correlated to fatigue development during stress, but was not correlated to pain or tension.

**Conclusion:**

Based on a lack of recovery of the blood pressure and the acral finger skin blood flow response to mental stress we conclude that these responses are more protracted than other physiological stress responses.

## Background

A substantial epidemiological literature has shown that mental and social stress is a risk factor for development of musculoskeletal pain, especially for pain in the shoulder and neck [1-4]. Different theoretical models for possible causal links between stress and health complaints have been described. Eriksen and Ursin [5] describe a process of psychological sensitisation and arousal leading to intolerable subjective complaints. McEwen and co-workers [6,7] describe a similar model with more emphasis on physiological responses, introducing the concept of allostatic load (i.e., the physiological result of chronic exposure to stress). The lack of physiological recovery after stress is considered by both groups a key factor linking stress and disease. Furthermore, laboratory studies indicate that autonomic activation and dysfunction is implicated in chronic pain [8]. In the search for possible biological correlates for the link between stress and disease, earlier laboratory studies have used short lasting stressors with analytical focus on the physiological reactivity (response to the stress), while the important physiological recovery period has received little attention [9]. Little is known about the physiology of the recovery period after stressful and repetitive work-related tasks.

In order to explore further the physiological basis for the link between stress and muscle pain, which again may relate to chronic pain development, we performed this study on healthy subjects performing a long-lasting stressful task (1 hour) with a 30 minute recovery period. We used a stressful task of sufficient duration to mimic real-world (e.g. work-related) stress, adding external validity to the methodology [10]. The stressful task has previously been used to explore the development of subjective complaints and muscular activity to stress in pain-free controls [11] and in patient groups with musculoskeletal pain or headache [12-15]. However, activity in the autonomic nervous system was not assessed in the previous studies. In the present study we measured muscle activity (surface electromyography) together with blood pressure, heart rate, acral finger skin blood flow and respiration frequency 10 minutes before, during the 60 minute stressful task and 30 minutes after. Development of pain, fatigue and tension was recorded immediately before and every 10 minutes during the stressful task and in the 30 minute rest period.

Firstly, we wanted to describe the autonomic and muscular response and recovery profiles after low-grade mental stress of long duration in healthy subjects. Secondly, we hypothesized that development of subjective complaints during a long lasting low-grade stressful task were related to the physiological response to the task. Lastly, we hypothesized that those variables with the slowest recovery profile would be related to the subjective complaints induced by the stressful task.

## Methods

### Subjects

Forty-four healthy subjects participated in the study (Table 1). The participants were recruited as controls for a group of pain patients with a female predominance, and therefore comprised thirty-five women and nine men. They were recruited from public institutions and private companies in Trondheim. Subjects were excluded if they fulfilled all of the three following criteria: (1) headache or musculoskeletal pain for more than one day per month, and (2) had visited a physician, and (3) took medication for the complaint (all three conditions to be fulfilled). In addition, subjects considering their headache or pain to be more than "unpleasant" (i.e. a higher degree of pain) were excluded if (1) they experienced the pain more than one day per month, or (2) had visited a physician for the pain, or (3) took medication for the pain (i.e. any of the three conditions fulfilled). No participants took drugs with a possible interaction with neural, vascular or muscular function (e.g. antiepileptics, β-blockers, and antidepressants).

**Table 1:** Subject characteristics for the 44 participants

	Mean (SD)	Range
Age all (n = 44, years)	41 (12)	21–61
Age women (n = 35, years)	40 (12)	21–61
Age men (n = 9, years)	37 (12)	19–56
Weight (kg)	72 (14)	47–103
Height (cm)	168 (8)	145–190
Autonomic symptom index	5 (3)	1–13
EPQ-N	7 (4)	0–15
		No. of subjects (%)
Married/cohabitant (n)		31 (71%)
Working (≥ 50%) (n)		38 (86%)
Regular exercisers (≥ 1 session pr. week) (n)		14 (32%)
Smokers (n)		12 (27%)
Drinking alcohol ≥ 2 days pr. week * (n)		9 (20%)

* One person drinking more than 3 days pr. week

### Procedure

All subjects answered a questionnaire on biographical data (marital status, weight, medication, and stimulants), exercise habits, and the neuroticism index of the Eysenck Personality Questionnaire (EPQ-N)[16]. The questionnaire further included an index of symptoms concerning the autonomic nervous system ("autonomic symptom index"). For this purpose a subset of ten questions were chosen (No. 26–35) from the Composite Autonomic Symptom Profile [17]. The questions assessed different domains of autonomic symptoms (orthostatic, sudomotor, gastrointestinal, visual, vasomotor, reflex syncope). Sub-indexing different autonomic domains was not done due to the limited number of questions. The answers were graded. A serious extent of a symptom was given a higher value than a less serious. E.g. the answer to the questions: "In the last year, to what extent have you been in a cold sweat?", were graded as: "have not had" (value 0), mild (value 1), moderate (value 2), severe (value 3). The highest possible sum score was 30.

All potential participants went through a short telephone interview to exclude those not fulfilling inclusion criteria. Subjects not excluded by the initial screening received the questionnaire by post within two weeks of the test day. On the morning of the test day the subjects first went through a short interview controlling the answers from the questionnaire. Afterwards venous blood was sampled from the right cubital fossa. Subjects were instructed to empty their bladder before starting the test. Brassieres were removed and subjects wore only a light shirt on the upper part of the body. The laboratory temperature was regulated to 24.5 ± 1.0°C and was recorded every ten minutes during the experiment.

The subject was seated in an office chair with the lower arms resting on the table top before, during and after the test. Subjects got acquainted to the work-task by performing a mini-trial with instructions before the test started. The mini-trial was performed without introducing stress-imposing feedback on reaction time and was used to determine the subjects' habitual, non-stressed reaction time. Short maximal voluntary contractions were performed on each pair of muscles twice (frontalis muscle – raising eyebrows, temporalis – clenching teeth, neck – pushing head back against resistance, trapezius – pushing extended arms upwards against resistance at 45° angle out from the body). The maximal contractions were carried out in order to normalize the muscle activity during test to a percent of maximal force. However, the variability between the two maximal muscle contractions in the frontalis muscle was too large to make a reliable estimate of the maximal muscle force and thus none of the muscle activity measurements were normalized. In order to measure the subjects habitual level of physiological activation, the laboratory experiment started with a five minute period which served as a baseline period for the physiological variables. The subjects were alone in the room and were not given any instructions other than to find a comfortable position with their arms resting on the table in front of them. To ensure that all subjects had the same low level of muscle activity before the test started a five minute feedback period with muscle activity visualized on a screen followed. The subject experienced how it was possible to influence the level of muscle activity by adopting different postures and thereafter concentrated on minimising any muscle activity. The stressful task [18] was then performed: a two-choice reaction-time test on a monitor, lasting one hour. An open ("frame") and a solid ("brick") quadrangle were placed in a square pattern, and a written suggestion on how to move the brick to superimpose on the frame was given. The subject responded by pressing one of two keys ("correct" or "wrong") with the right middle or index finger. The task was to be carried out as quickly and correctly as possible. The PC program provided feedback on whether an answer was correct or wrong, and on the response time (very slow, slow, normal, fast, very fast) related to the subjects performance in the mini-trial carried out before the experiment started. Together with the feedback a new task was presented. After the end of the stressful task, all measurements continued for thirty minutes. The test person was instructed to sit still and relax during the rest period. Pain, perceived tension and fatigue was reported immediately before (baseline) and every ten minutes during and after the test by scoring on a 100 mm visual analogue scale (VAS) with the endpoints marked no pain/tension/fatigue and worst imaginable pain/tension/fatigue. The subjects were asked to assess pain in locations corresponding to the electromyography electrode positions; in the shoulders, neck, temples and forehead on both sides. The subjects were not allowed to see previous records when scoring.

A second blood sample was drawn during 5 min immediately after the test, before the 30 minute recovery period. Blood analysis was not a major aim of the study and these results are reported elsewhere (Nilsen et al., submitted).

### Physiological recordings

Muscle activity was quantified by bilateral bipolar recording of surface electromyography (SEMG) (electrode diameter 6 mm, inter-electrode distance 20 mm). The system noise level was less than 1.5 μV root mean square (RMS). The signals were bandpass-filtered (10–1250 Hz) and stored on a digitizing recorder (Earth Data 128). Data were subsequently fed into an A/D converter (Powerlab 16S; ADInstruments Pty Ltd, Sydney, Australia; sampling rate 2 kHz) for calculation of the RMS values (100 ms running time window). Sharp transients and electrical activity from the heart in the SEMG signals were removed with a median filter (Matlab ver 6, The MathWorks inc.).

The following electrode sites were used: (1) Frontalis muscle; both electrodes placed on a vertical line crossing the pupil, 10 mm and 30 mm above the upper border of the eyebrow. (2) Temporal muscle; the lower electrode 10 mm posterior to the lateral canthus of the orbit, and the second electrode 20 mm above. (3) Splenius muscle; upper electrode 35 mm lateral to the spinous process of C2, and the second electrode 20 mm below. (4) Trapezius muscle; medial electrode 10 mm lateral to the midpoint of a line connecting the acromion and the spinous process of C7, and the second electrode 20 mm lateral to the first electrode. The ground electrode was placed on the spinous process of C7.

Activity in the autonomic nervous system was assessed by measurements of continuous non-invasive finger blood pressure (Portapres)[19], measurements of skin blood flow with Laser-Doppler flowmetry (Moorlab, 4 channels, time constant 0.02 s, low-pass filter 22 kHz), and measurements of the respiration pattern with a thermistor (Flaga, Embla S-AF-010) below the nose with active elements in each nostril and in front of the mouth. The blood pressure cuffs were mounted on the intermediate phalanx at the left middle and ring fingers. Finger skin blood flow was measured bilaterally with the electrodes (fibre separation 0.5 mm) placed on the volar side of the distal phalanx (pulp) of the thumb. Signals were sampled at 200 Hz.

Respiration frequency was calculated by the Chart 4.2 software (ADInstruments Pty Ltd, Sydney, Australia). Heart rate and blood pressure were calculated with the Beatscope 1.0 software (TNO, Amsterdam, the Netherlands). One blood pressure recording could not be analyzed due to technical difficulties.

Technical difficulties resulted in exclusion of seven subjects from analysis of respiration frequency and exclusion of two subjects from analysis of heart rate and blood pressure responses.

### Analysis and statistics

Mean values for each 10-minute period were calculated for all physiological recordings. Muscular activity and finger blood flow values are reported as the average of the left and right side for each region because ANOVA repeated measures analysis (rANOVA) revealed no side differences for the finger skin blood flow and muscle activity except for the frontalis muscle SEMG (left side (10.9 μV) > right side (9.2 μV); F(43) = 8.0, p = 0.007). However, performing all subsequent tests separately for right and left frontalis muscles did not give deviant results from those reported. Pain scores are reported from the side with the highest response (there were no side differences in neither pain level (side effect) nor pain development (side × time effect) for any of the four regions (rANOVA, Fs ≤ 3.2, p ≥ 0.08).

ANOVA with repeated measurements was used for evaluation of subgroup effects (sex, marital status, employment status, regular exercise, smokers, and alcohol drinking introduced sequentially one at a time as between-subject factors) with ten time intervals. For subgroup analysis of the recovery period we calculated a recovery variable (the difference between the mean of the last 10 minutes of rest (85–95 min) and the baseline period mean), a measure considered to be more meaningful than the absolute level when comparing groups [9]. Feedback data is displayed in figures, but feedback was not included in ANOVAs because we intended to study responses related to stress in this study. Recovery variables were analysed with one-way ANOVA tests.

For evaluation of the total response to the test we first performed repeated measures ANOVA tests (no between-subjects factors, evaluating the within-subject effect of time) with the same time intervals as in the subgroup analysis. For further post-hoc exploration of the response and recovery time-course we performed a series of paired-sample tests (Student's t-tests for physiological variables (Gauss-distributed) and Wilcoxon signed rank test for subjective variables (not Gauss-distributed)): We first evaluated the early response to the stressful task by comparing the first part (0–10 min) of the stressful task to baseline (immediately before the stressful task for the subjective variables). Secondly, changes during the stressful task (adaptation/summation effects) were investigated by a comparison of the first (0–10 min) and the last (50–60 min) part of the stressful task. Thirdly, we evaluated the recovery by comparing the change from the end of the stressful task (50–60 min) with the first part of the recovery period (65–75 min) and the first (65–75 min) and last (85–95 min) part of the recovery period with baseline.

Physiological responses (the difference between the average of the whole stressful task (0–60 min) and the average of the baseline period) and subjective responses to the stressful task (the difference between the maximal value during the 60 minute stress period and the value reported immediately before starting the test) were calculated as summary-variables for correlation analysis. Subjects with a pain response larger than zero were defined as pain responders. For each subject the location with the highest pain response during the task was identified (i.e. only one location for each subject). The pain response in this location (maximal pain location) was treated as a separate summary variable in the analysis (and it is the pain scores in this specific location we have displayed graphically).

Possible associations between variables were investigated by correlating the muscular responses (trapezius, splenius, temporalis, frontalis) with the autonomic responses (systolic and diastolic blood pressure, heart rate, respiration frequency and finger skin blood flow), and by correlating physiological responses (as above) with subjective responses (maximal pain, tension and fatigue), and finally by correlating the subjective responses with each other (i.e. maximal pain, tension and fatigue). The correlation coefficients between pain and muscular responses were calculated separately for each localisation (i.e. left temple pain with left temporalis muscle activity). Furthermore, as post-hoc analysis we searched for possible correlations between blood pressure/finger skin blood flow recovery variables and physiological responses, subjective responses and other recovery variables. We used Pearson correlation (r_p_) for physiological variables (Gauss-distributed) and Spearman's rank order correlation (r_s_) when subjective data were involved (not Gauss-distributed).

Because Mauchly's test of sphericity was significant in all ANOVA repeated measures tests with time as a within-subject effect we used Huynh-Feldt correction of degrees of freedom for these results. Two-tailed p-values less than 0.05 were considered to be significant. Because the hypotheses testing in this study involved several autonomic subsystems with insufficient a priori knowledge on possible relation to pain, we did not correct for multiple comparisons.

### Ethics

For transport expenses and the inconvenience (total time expenditure for each participant was 4 hours) participants received NOK 500 (USD 75). The Regional Committee for Medical Ethics approved the protocol, and all participants gave written informed consent before volunteering. Experiments were performed according to the Helsinki Declaration.

## Results

All variables are listed in Table 2 with the results of the paired comparisons summarised in Table 3.

**Table 2:** Mean values and the average responses for all variables

	Baseline	During the stressful task						Response*
Variable	Mean (SD)	0–10 min Mean (SD)	10–20 min Mean (SD)	20–30 min Mean (SD)	30–40 min Mean (SD)	40–50 min Mean (SD)	50–60 min Mean (SD)	Mean (SD)
Surface electromyography								
Trapezius (μV)	6.2 (6.2)	11.8 (13.7)	12.0 (12.8)	11.6 (13.8)	10.5 (11.3)	11.2 (12.4)	10.7 (11.8)	5.1 (11.4)
Splenius (μV)	5.3 (3.2)	4.7 (2.7)	4.6 (2.3)	4.6 (2.6)	4.5 (3.0)	4.6 (3.5)	4.6 (3.1)	-0.7 (3.1)
Temporalis (μV)	6.5 (3.2)	6.4 (4.7)	7.0 (6.1)	6.6 (5.6)	7.1 (5.2)	7.3 (5.5)	7.2 (5.1)	0.5 (5.4)
Frontalis (μV)	8.0 (5.9)	11.1 (5.8)	11.1 (6.1)	11.0 (6.6)	11.3 (6.4)	11.1 (6.5)	11.4 (6.5)	3.2 (4.8)
Systolic BP (mmHg)	112 (16)	126 (17)	122 (16)	122 (15)	123 (15)	123 (15)	125 (15)	11.4 (7.8)
Diastolic BP (mmHg)	62 (11)	72 (13)	69 (13)	69 (11)	71 (12)	70 (11)	71 (10)	8.6 (5.0)
Heart rate (beats/min)	71 (8)	75 (10)	74 (9)	73 (9)	72 (9)	72 (9)	72 (8)	2.3 (4.3)
Respiration (breaths/min)	15 (3)	17 (3)	17 (3)	16 (3)	16 (3)	16 (3)	16 (3)	1.5 (2.5)
Skin blood flow (au)	279 (112)	248 (122)	251 (130)	246 (127)	249 (126)	237 (127)	229 (120)	-35.3 (56.7)
Pain (VAS 0–100 mm)		10 min	20 min	30 min	40 min	50 min	60 min	
Maximal location (mm)	2.4 (6.1)	3.0 (5.3)	3.6 (5.8)	6.7 (11.1)	9.0 (14.4)	11.9 (15.5)	14.0 (17.1)	15.4 (18.0)
Shoulder (mm)	2.8 (6.3)	2.4 (5.6)	4.5 (7.0)	5.4 (8.8)	6.5 (12.4)	8.7 (13.3)	10.3 (14.4)	12.9 (16.1)
Neck (mm)	2.4 (5.1)	3.1 (5.6)	3.5 (6.4)	5.0 (9.1)	6.4 (8.2)	8.6 (12.2)	9.8 (11.9)	11.5 (13.7)
Temples (mm)	1.0 (2.2)	2.3 (5.2)	1.9 (4.1)	3.7 (8.2)	4.2 (9.1)	5.6 (10.8)	5.5 (11.7)	7.5 (13.7)
Forehead (mm)	1.1 (2.5)	1.6 (3.3)	2.5 (6.0)	3.8 (8.8)	4.2 (9.1)	4.7 (9.5)	5.3 (11.1)	6.3 (12.0)
Fatigue (VAS 0–100 mm)	8.9 (15.3)	7.7 (13.5)	10.8 (14.6)	19.0 (20.5)	22.2 (21.2)	29.8 (22.5)	33.1 (25.4)	27.2 (23.1)
Tension (VAS 0–100 mm)	7.0 (12.4)	11.2 (13.2)	12.9 (14.0)	18.0 (19.1)	19.4 (20.2)	21.7 (20.6)	25.3 (23.1)	21.2 (21.2)

		Recovery period			Recovery §			
Variable		65–75 min	75–85 min	85–95 min				

Surface electromyography								
Trapezius (μV)		5.4 (4.7)	5.6 (4.5)	6.2 (6.4)	0.14 (7.7)			
Splenius (μV)		4.7 (2.7)	4.9 (3.0)	4.9 (3.4)	-0.33 (3.6)			
Temporalis (μV)		7.7 (4.9)	7.4 (5.3)	7.1 (4.2)	0.68 (3.8)			
Frontalis (μV)		9.0 (6.9)	8.2 (5.5)	8.3 (5.1)	0.26 (3.9)			
Systolic BP (mmHg)		123 (15)	122 (14)	124 (14)	12.1 (11.7)			
Diastolic BP (mmHg)		71 (10)	69 (9)	71 (10)	9.5 (6.6)			
Heart rate (beats/min)		69 (7)	69 (8)	69 (8)	-1.9 (3.8)			
Respiration (breaths/min)		14 (2)	14 (2)	14 (3)	-0.68 (2.7)			
Skin blood flow (au)		215 (105)	229 (111)	211 (106)	-67.5 (89.1)			
Pain (VAS 0–100 mm)		75 min	85 min	95 min				
Maximal location (mm)		6.9 (15.2)	7.6 (14.9)	5.8 (13.5)	3.3 (11.4)			
Shoulder (mm)		6.2 (15.0)	6.2 (14.9)	5.3 (13.7)	2.5 (11.4)			
Neck (mm)		5.5 (11.0)	6.1 (12.5)	5.5 (11.5)	3.0 (10.1)			
Temples (mm)		1.9 (5.9)	1.7 (4.5)	1.7 (5.2)	0.67 (4.7)			
Forehead (mm)		2.2 (6.1)	1.8 (4.5)	1.8 (5.1)	0.63 (4.6)			
Fatigue (VAS 0–100 mm)		17.2 (20.2)	17.5 (21.8)	15.0 (20.2)	6.7 (19.3)			
Tension (VAS 0–100 mm)		8.2 (16.0)	8.1 (16.5)	4.8 (12.0)	-1.4 (12.0)			

au: arbitrary units. BP: blood pressure

* Response = Average during stressful task (0–60 min) – baseline for the physiological variables, and maximal during stressful task (0–60 min) – baseline for the subjective variables.

§Recovery = The last ten minutes of the recovery period (85–95 min) – baseline (summary statistics used in correlation analysis).

**Table 3:** Test statistics for evaluation of the response and recovery to the stressful task

Variable	rANOVA*	Baseline vs. 0–10 min	0–10 vs. 50–60 min	50–60 vs. 65–75 min
Surface electromyography				
Trapezius (μV)	F(2.5,105.6) = 7.3, p < 0.001	t(43) = -2.9, p = 0.006	t(43) = 1.0, p = 0.33	t(43) = 3.1, p = 0.003
Splenius (μV)	F(3.3,144.0) = 0.96, p = 0.42	t(43) = 1.1, p = 0.28	t(43) = 0.5, p = 0.62	t(43) = -0.2, p = 0.80
Temporalis (μV)	F(2.7,117.8) = 1.2, p = 0.31	t(43) = 0.0, p = 0.96	t(43) = -2.3, p = 0.03	t(43) = -0.9, p = 0.39
Frontalis (μV)	F(3.2,138.2) = 11.3, p < 0.001	t43) = -3.8, p < 0.001	t(43) = -0.4, p = 0.69	t(43) = 3.3, p = 0.002
Systolic blood pressure (mmHg)	F(2.8,14.3) = 11.4, p < 0.001	t(40) = -7.0, p < 0.001	t(40) = 0.3, p = 0.78	t(41) = 1.5, p = 0.15
Diastolic blood pressure (mmHg)	F(3.0,118.2) = 17.4, p < 0.001	t(40) = -7.6, p < 0.001	t(40) = 0.4, p = 0.66	t(41) = 0.1, p = 0.96
Heart rate (beats/min)	F(2.6,103.2) = 24.1, p < 0.001	t(40) = -4.5, p < 0.001	t(42) = 3.7, p = 0.001	t(42) = 8.1, p < 0.001
Respiration (breaths/min)	F(4.3,155.2) = 21.7, p < 0.001	t(36) = -4.6, p < 0.001	t(36) = 2.1, p = 0.04	t(36) = 6.1, p < 0.001
Finger skin blood flow (au)	F(2.6,113.2) = 6.2, p < 0.001	t(43) = 3.6, p = 0.001	t(43) = 1.8, p = 0.09	t(43) = 1.6, p = 0.12
Pain (VAS 0–100 mm)				
Maximal location (mm)	F(3.2,139.4) = 7.8, p < 0.001	Z = -1.4, p = 0.17	Z = -4.6, p < 0.001	Z = -4.0, p < 0.001
Shoulder (mm)	F(2.7,112.5) = 3.8, p = 0.02	Z = -0.3, p = 0.75	Z = -4.3, p < 0.001	Z = -3.5, p < 0.001
Neck (mm)	F(2.4,98.9) = 4.5, p = 0.01	Z = -0.6, p = 0.52	Z = -4.3, p < 0.001	Z = -3.2, p = 0.001
Temples (mm)	F(2.0,86.5) = 4.1, p = 0.02	Z = -2.2, p = 0.03	Z = -1.9, p = 0.06	Z = -2.7, p = 0.006
Forehead (mm)	F(2.1,89.5) = 4.0, p = 0.02	Z = -2.5, p = 0.01	Z = -2.7, p = 0.008	Z = -2.7, p = 0.008
Fatigue (VAS 0–100 mm)	F(3.2,129.0) = 17.0, p < 0.001	Z = -0.6, p = 0.52	Z = -5.2, p < 0.001	Z = -4.4, p < 0.001
Tension (VAS 0–100 mm)	F(2.6,104.7) = 16.1, p < 0.001	Z = -2.4, p = 0.02	Z = -4.5, p < 0.001	Z = -4.9, p < 0.001

		50–60 vs. 85–95 min	Baseline vs. 65–75 min	Baseline vs. 85–95 min

Surface electromyography				
Trapezius (μV)		t(43) = 2.8, p = 0.008	t(43) = 0.9, p = 0.40	t(43) = -0.12, p = 0.99
Splenius (μV)		t(43) = -7.1, p = 0.48	t(43) = 1.4, p = 0.16	t(43) = 0.65, p = 0.54
Temporalis (μV)		t(43) = 0.2, p = 0.83	t(43) = -2.0, p = 0.05	t(43) = -1.2, p = 0.24
Frontalis (μV)		t(43) = 4.5, p < 0.001	t(43) = -1.7, p = 0.10	t(43) = -0.44, p = 0.67
Systolic blood pressure (mmHg)		t(41) = 0.54, p = 0.60	t(40) = -7.1, p < 0.001	t(40) = -6.5, p < 0.001
Diastolic blood pressure (mmHg)		t(41) = -0.4, p = 0.69	t(40) = -9.5, p < 0.001	t(40) = -9.2, p < 0.001
Heart rate (beats/min)		t(42) = 7.0, p < 0.001	t(40) = 3.1, p = 0.004	t(40) = 3.2, p = 0.003
Respiration (breaths/min)		t(36) = 5.6, p < 0.001	t(36) = 2.2, p = 0.03	t(36) = 1.5, p = 0.14
Finger skin blood flow (au)		t(43) = 1.7, p = 0.10	t(43) = 5.4, p < 0.001	t(43) = 5.0, p < 0.001
Pain (VAS 0–100 mm)		60 vs. 95 min	0 vs. 75 min	0 vs. 95 min
Maximal location (mm)		Z = -4.0, p < 0.001	Z = -2.5, p = 0.01	Z = -2.4, p = 0.015
Shoulder (mm)		Z = -3.5, p < 0.001	Z = -1.7, p = 0.08	Z = -1.3, p = 0.20
Neck (mm)		Z = -2.7, p = 0.007	Z = -2.1, p = 0.04	Z = -2.2, p = 0.03
Temples (mm)		Z = -2.7, p = 0.007	Z = -0.4, p = 0.72	Z = -0.51, p = 0.61
Forehead (mm)		Z = -2.7, p = 0.007	Z = -0.7, p = 0.48	Z = -0.40, p = 0.69
Fatigue (VAS 0–100 mm)		Z = -4.4, p < 0.001	Z = -3.2, p = 0.001	Z = -2.6, p = 0.009
Tension (VAS 0–100 mm)		Z = -4.8, p < 0.001	Z = -0.3, p = 0.78	Z = -1.5, p = 0.13

au: arbitrary units. BP: blood pressure

* ANOVA repeated measures (no between-subjects factors, time effect) with ten time intervals (baseline, 0–10, .., 85–95 min) and Huynh-Feldt corrected degrees of freedom. All other statistics are paired statistics (Student's paired t-tests for physiological variables and Wilcoxon paired statistics for subjective variables used in explorative contrast analysis).

### Physiological responses

**Figure 1:**
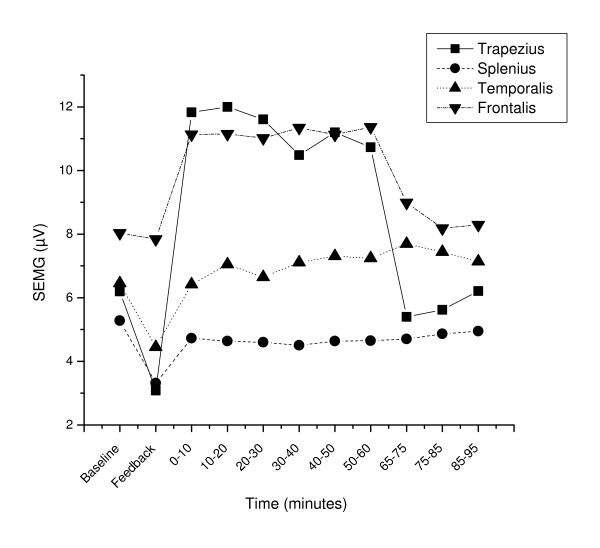
Surface electromyographic (SEMG) activitity before (Baseline, Feedback), during (0–10, 10–20, .., 50–60 min) and after (65–75, 75–85, 85–95 min) the stressful task. Mean RMS values for periods of 10 minutes (Baseline, Feedback: 5 min) are shown.

**Figure 2:**
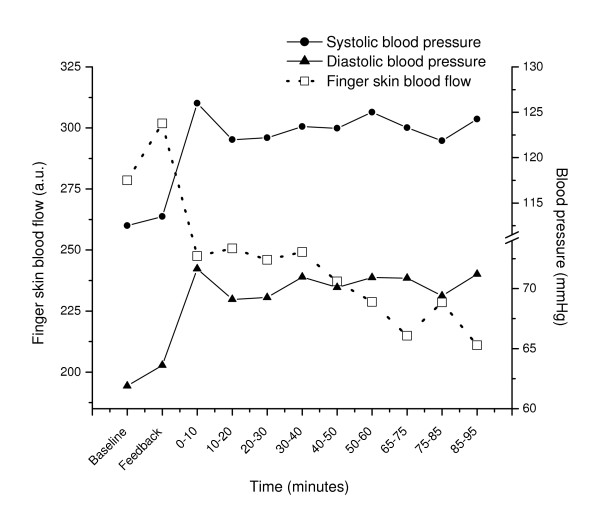
Mean blood pressure and finger skin blood flow (SBF) before (Baseline, Feedback), during (0–10, 10–20, .., 50–60 min) and after (65–75, 75–85, 85–95 min) the stressful task. Mean values for periods of 10 minutes (Baseline, Feedback: 5 min) are shown. Au = arbitrary units.

**Figure 3:**
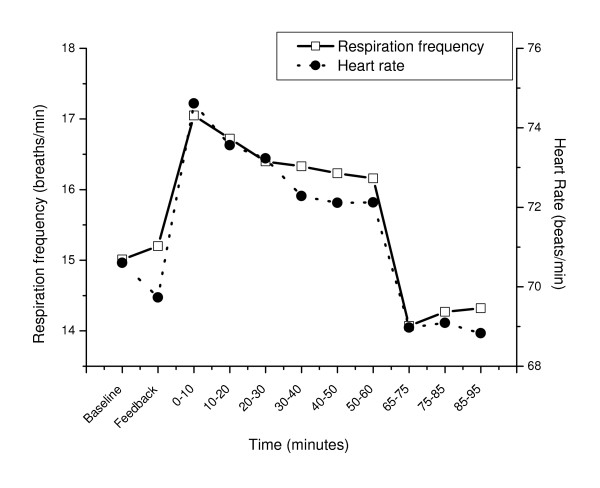
Respiration frequency and heart rate before (Baseline, Feedback), during (0–10, 10–20, .., 50–60 min) and after (65–75, 75–85, 85–95 min) the stressful task. Mean values for periods of 10 minutes (Baseline, Feedback: 5 min) are shown.

The development of all physiological variables is illustrated in Figure 1, 2 and 3.

The stressful task induced a clear response evident in all physiological variables (Table 2 and 3; baseline vs. 0–10 min, p ≤ 0.006) except for the splenius (p = 0.28) and temporalis muscle SEMG (p = 0.96).

Furthermore, age correlated negatively with the average respiration frequency response (r_p _= -0.44, p = 0.006) and height correlated negatively with the average systolic blood pressure response (r_p _= -0.41, p = 0.008). None of the other physiological responses (Table 2) correlated with age, height or weight.

Comparing the last ten minutes of the stressful task to the first ten minutes of the stressful task (Table 2 and 3) revealed a fall in heart rate with 2.5 beats/min (p = 0.001) and a reduced respiration frequency with 0.89 breaths/min (p = 0.04), indicating adaptation to the task for these two variables only. However, in the same time interval temporalis muscle activity increased with 0.82 μV (p = 0.03) and finger skin blood flow showed a trend towards lower values (p = 0.09). The other physiological variables were stable throughout the stressful task (p ≥ 0.33).

The heart rate response correlated with the trapezius muscle response (r_p _= 0.44, p = 0.004) and the temporalis muscle response (temporalis vs. heart rate, r_p _= 0.41, p = 0.008). The other correlations in the SEMG vs autonomic response matrix were non-significant (p > 0.06).

### Physiological recovery

Upon cessation of the stressful task, heart rate (p < 0.001), respiration frequency (p < 0.001) and muscle activity in the trapezius (p < 0.003) and the frontalis (p < 0.002) decreased significantly (50–60 min vs. 65–75 min). Trapezius and frontalis SEMG recovered to the baseline level (baseline vs. 65–75 min, p ≥ 0.10) while heart rate and respiration frequency recovered to a level lower than baseline (baseline vs. 65–75 min, p ≤ 0.03). However, systolic and diastolic blood pressure, finger skin blood flow and muscle activity in the splenius and temporalis muscles did not change significantly upon cessation of the stressful task (50–60 min vs. 65–75 min and 50–60 min vs. 85–95 min, p > 0.10). The systolic and diastolic blood pressure level remained elevated and finger skin blood flow was reduced during the whole recovery period (baseline vs. 85–95 min p ≤ 0.001).

The finger skin blood flow recovery variable (Table 2) correlated negatively the systolic and diastolic blood pressure recovery variables (r_p _= -0.52, p = 0.001 and r_p _= -0.40, p = 0.01 respectively). This means that a high blood pressure at the end of the recovery period was associated with a small finger skin blood flow at the same time. The finger skin blood flow and blood pressure recovery variables did not correlate with other physiological (HR, muscle, respiration) response or recovery variables (r ≤ 0.25, p ≥ 0.11).

### Subjective responses and recovery

**Figure 4:**
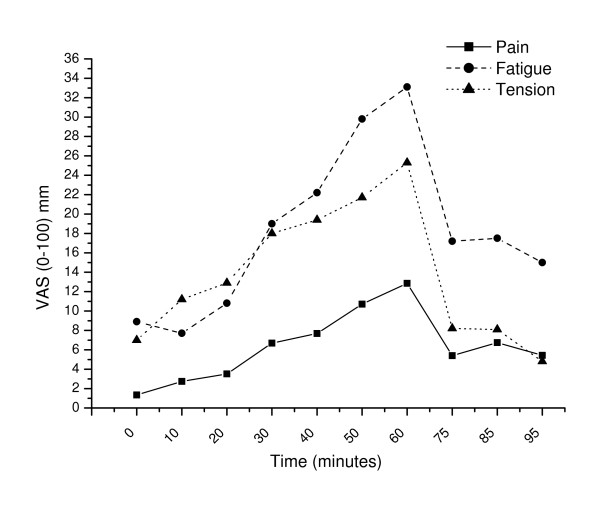
Tension, fatigue and pain scores in the maximal pain location before (0 min), during (10, 20, .., 60 min) and after (75, 85, 95 min) the stressful task.

Development of tension, fatigue and pain scores in the maximal pain location is illustrated in Figure 4. Subjects reported increased tension (p = 0.02) and increased pain in the temples (p = 0.03) and forehead (p = 0.01) already ten minutes into the stressful task (0 min vs. 10 min), while fatigue (p = 0.52) and pain in the shoulder and neck (p > 0.52) did not increase during the first ten minutes (Table 2 and 3). All subjective variables increased further during the stressful task (10 min vs. 60 min; p < 0.008 except for a trend in temple pain (p = 0.06)), and were significantly reduced ten minutes into the recovery period (60 min vs. 75 min, p < 0.008). However, fatigue and pain in neck (and maximal pain) did not recover to baseline (0 min vs. 95 min; p < 0.04). Pain in the shoulders showed a trend towards non-recovery ten minutes into the recovery period (p = 0.08) but recovered to baseline after 30 minutes (p = 0.20), while tension and pain in temples and forehead returned to baseline ten minutes into the recovery period (p > 0.48).

Thirty subjects (68.2%) reported an increase in pain in at least one location during the test and twenty-eight subjects (63.6%) had an increase in pain VAS score of more than 30 mm during the test (Table 4). The pain response was most evident in the neck and/or shoulder (Table 4).

**Table 4:** Subjective responses categorized in three groups

	VAS = 0	VAS 1–30	VAS > 30
Pain:			
Shoulders (n (%))	17 (38.6%)	3 (6.8%)	24 (54.5%)
Neck (n (%))	19 (43.2%)	1 (2.3%)	24 (54.5%)
Temples (n (%))	27 (61.4%)	5 (11.4%)	12 (27.3%)
Forehead (n (%))	24 (54.5%)	8 (18.2%)	12 (27.3%)
Maximal pain location^b^	14 (31.8%)*	2 (4.5%)	28 (63.6%)
Fatigue (n (%))	5 (11.6%)	1 (2.3%)	37 (86.0%)
Tension (n (%))	4 (9.5%)	3 (7.1%)	35 (83.3%)

^a ^Pain response = (maximal pain during test – pain before test), ^b ^Maximal pain response irrespective of location,* = No pain development in any location.

Pain responses did not correlate with tension and fatigue responses (r_s _≤ 0.19, p ≥ 0.20), however, fatigue and tension responses were correlated (r_s _= 0.48, p = 0.001).

**Figure 5:**
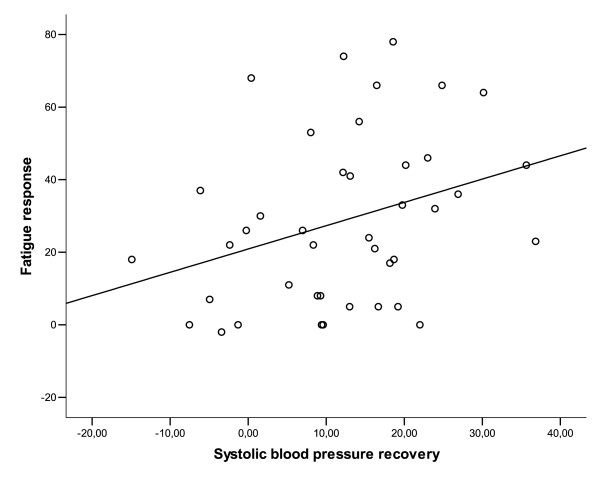
Blood pressure recovery (value at 95 min – baseline) plotted against the fatigue response with linear regression line shown. The association is significant (r_s _= 0.34, p = 0.03).

Pain, tension and fatigue responses did not correlate significantly with physiological responses (r_s _≤ 0.28, p ≥ 0.071, correlation coefficients between pain and muscular responses were calculated separately for each localisation). However, the fatigue response correlated with systolic (r_s _= 0.34, p = 0.03, Figure 5) and diastolic blood pressure recovery (r_s _= 0.31, p = 0.047) indicating a larger fatigue response during the stressful task for those subjects who recovered less during the rest period. However, no significant correlations were found between the blood pressure recovery and the pain and tension response variables (r_s _≤ 0.16, p ≥ 0.31) and finger skin blood flow recovery was not correlated to subjective responses (r_s _≤ 0.16, p ≥ 0.29).

Except for a correlation between the autonomic symptom index (Table 1) and the blood pressure response (Table 2, r_s _= 0.38, p = 0.014), the physiological responses were not correlated to the Nevroticism index or the "autonomic symptom index". The Nevroticism index (EPQ-N, Table 1) correlated with pain and fatigue responses (r_s _≥ 0.36, p ≤ 0.016).

### Subgroup analyses

Subgroup analyses with the dichotomized variables in Table 1 (sex, marital status, employment status, regular exercisers, smokers, and alcohol drinking) revealed that women had lower respiratory frequency (15.2 vs. 17.1 breaths/min, rANOVA; sex effect F(1,35) = 4.5, p = 0.04) and higher frontalis SEMG (11.1 vs. 6.1 μV, rANOVA; sex effect F(1,42) = 6.7, p = 0.01). Moreover, smokers had higher blood systolic blood pressure level (130 mmHg vs. 120 mmHg, rANOVA; smoking effect F (1,36) = 4.7, p = 0.04) and we found a time × marital status interaction for maximal pain (rANOVA; F(3.0,141.2) = 2.6, p = 0.048) with higher maximal pain response for those living alone compared to cohabitants (17.7 vs 14.5 mm VAS). Subgroup analysis of the recovery variables did however not reveal any differences (One-way ANOVA Fs ≤ 2.9, p ≥ 0.097). It must be noted that some subgroups had few cases (Table 1), and were not ideal for subgroup effect analysis.

## Discussion

Major findings in the present study on stress responses in healthy subjects can be summarized as: 1) A significant proportion of healthy subjects (64%) respond with a pain increase of more than 30 mm (VAS 0–100) in at least one of the four muscle groups investigated. 2) Pain develops gradually as a response to a stressful task. 3) The trapezius and frontalis muscles are activated in response to the task with fast recovery after a stressful task. 4) The HR-response habituates gradually during a long-lasting stressful task and recovers fully afterwards. 5) There is a lack of skin blood flow and blood pressure recovery after a stressful task of long duration. 6) Physiological responses (and recovery) are not correlated with pain responses, but 7) lack of blood pressure recovery is correlated to the fatigue response to the preceding stressful task.

The most important finding is that blood pressure and finger skin blood flow did not recover to baseline during the 30-min rest period, contrasting the recovery pattern of the other autonomic and muscular responses. The finger skin blood flow apparently had biphasic response pattern with a fast reduction during the first ten minutes of the stressful task and a further monotonic reduction (trend) during the stressful task, while the blood pressure increased during the first ten minutes of the stressful task and stayed elevated both during and after the stressful task.

Slow recovery of blood pressure following experimental stress has previously been reported by Steptoe and co-workers [20,21]. They applied the colour-word test and mirror tracing for a total stress period of 10 min, causing a stress response marginally higher than in the present series, judged by the increase in heart rate (Δheart rate ~7 vs. 5 bpm) and blood pressure (Δblood pressure ~14 vs. 11 mmHg). In their study blood pressure had partially recovered 20–25 min after the test (female subjects) while in the present study no recovery was observed after 30 min. Assuming a similar level of stress in the two series, the slower time course of blood pressure recovery in the present study can be due to the longer duration of the stress period.

The present study of healthy controls shows that slow vascular recovery after mental stress is a normal phenomenon and is not related to simultaneous pain development. The theoretical models linking stress and subjective health complaints emphasize lack of recovery after stress as an important factor for development of subjective complaints [5-7]. According to these models, a person with a reduced ability to recover after stress is more prone to develop subjective complaints. However, as the present study illustrates, when re-examining these theoretical models in the laboratory one may have to register physiological variables over longer recovery periods than we have done in our study to be able do detect possible differences in physiological recovery between patients and healthy controls.

A long-lasting, presumably sympathetically mediated vasoconstriction is evident in the present study. Environmental temperature was monitored throughout the experiment and was stable and not related to skin blood flow (data not shown). The slightly different time course of blood pressure and the finger skin blood flow response indicate differential control of vascular beds. This is interpreted as an example of the specificity of different neuroanatomical circuits within the autonomic nervous system [22] and corresponding differentiation of sympathetic responses with respect to target organ and response localisation within the vascular system [23-25].

Although the reduction of finger skin blood flow was not related to subjective complaints in the present study, it is potentially relevant that some patients with musculoskeletal complaints report a cold feeling in wrist/hand [26,27]. A recent study, using infrared thermography to measure dorsal hand skin temperature, showed that post-exercise hyperaemia was blunted in patients with chronic upper extremity pain who reported cold hands induced by keyboard use [28].

A previous study on pain-free subjects using a similar protocol, but without measurements of blood pressure, heart rate and respiration frequency, found a correlation between pain development and muscle activity in the right trapezius muscle (r = 0.37, p < 0.03) during the stressful task [11]. In the present study we found no correlation between pain response and muscle activity. Because the protocols were so similar and the study group were larger in the present study (44 subjects in the present study, 36 subjects in the previous study), we believe the different finding in the present study indicate that the earlier reported correlation may have been a chance finding. In the previous study increased muscular activity during the stressful task was found for the frontalis muscle and for the trapezius muscles (significant for the frontalis and a trend for the trapezius muscles), whereas no response to the stressful task was found for the splenius and temporalis muscles. The present study confirms the earlier findings of the frontalis and trapezius muscles as more responsive to a stressful task than the splenius and temporalis muscles.

In the present study most cardiovascular vs. EMG correlations were not significant. However, we found correlations between the heart rate response and trapezius and temporalis muscle responses. The correlations were strong (p < 0.005), and we cannot exclude that it is relevant. The electrical activity from the heart was filtered out of the electromyographic signals, and a correlation with heart rate was not observed for the splenius muscles, hence it is probably not related to an ECG-artefact. Increased muscular activity in a rather large muscle like trapezius is reasonably paralleled by increased HR if the increased muscular activity demands a higher cardiac output to satisfy the metabolic needs.

The low-grade stress response in the present experiments is shown by the heart rate only being elevated by 4 beats per minute (bpm) on average, and 5 bpm the first 10 min. Other studies of stress responses have exposed subjects to stress for a shorter period of time and report elevated heart rate responses of 10–20 bpm indicating a higher level of stress [29-31]. The pain reported in the present study is indeed low-level and not directly comparable to laboratory studies of acute pain. The level of tension and fatigue was considerably higher than the pain level in the present study. However, the levels of pain, tension and fatigue obtained in this laboratory study corresponds well with the values obtained from healthy subjects in field studies of workers in stressful work situations with low biomechanical load [32,33]. Therefore, we believe that the level of subjective complaints reported in this laboratory study is comparable to the subjective complaints healthy subjects experience during stressful and repetitive office work, although laboratory experiments never can substitute real-life experiments. Extending the duration of the stress exposure (as we have done in the present study) has been suggested as one way to increase the external validity of studies on cardiovascular responses to stress [10].

The subject's perception of the stressor was not considered in terms of stress level in the present study, but evaluated using the term "tension". Holte et al. [34] investigated the concept of tension in Norwegian subjects with questionnaires and qualitative interviews and found that subjects described tension in terms of both stress-related autonomic symptoms and musculoskeletal activation (the Norwegian word for tension ("anspenthet") conveys almost the same meaning as the word stress). Furthermore, different perception of the stressor may partly explain the large inter-subject variation in physiological responses [35,36]. Moreover, the lack of association between pain and tension responses may indicate that the pain is linked to physiological factors and not to cognitive factors alone.

The feedback period was necessary in order to ensure that all subjects had the same low level of muscle activity before the stressful task. The feedback was given solely on muscular activity. The feedback was introduced after the baseline period in order to get a true baseline period without influence from the feedback procedure. It is possible that the feedback procedure influenced the measured muscle activity during the stressful task by teaching the subjects how to relax their muscles. However, this effect was supposedly similar for all subjects. Furthermore, subjects did not receive any feedback on the measured variables during the stressful task.

In the correlation analysis we have used summary variables in order to minimize the number of calculated correlations. While the subjective variables were steadily increasing through the task, most physiological responses were more stable (although not without exceptions). The physiological variables were measured continuously and we did not want to place emphasis on any (possible random) peak value. Instead, the average value was considered a summary variable reflecting the total physiological "burden" of the stressful task. However, the average pain score will in our opinion not reflect the subjective "burden" of the stressful task. An average pain score would underestimate the pain-inducing effect of the stressful task in case the subject's pain pathways would have been sensitised in any way, thus potentially neglecting the effect of any temporal summation of pain. We have chosen to use the maximal value during the task as an approximation of this "burden", and this is in line with others [37-39].

Our subgroup analysis did not reveal any differences between groups regarding the recovery variables, and the present study thus confirms the findings of Steptoe [20] who reported no relationship between prolonged cardiovascular stress responses and sedentary lifestyle. We have not found any other studies related to our findings of lower respiration frequency and higher frontalis muscle activity in women. Considering that smoking is well-known risk factor for cardiovascular disease [40] our finding of increased blood pressure among smokers is not surprising. The higher pain response found for those living alone is very difficult to explain and we are not aware of any other study who has investigated this. However, as already noted, subgroup sizes were partly asymmetric and not optimally sensitive for subgroup factor effect analysis.

We are not aware of other studies investigating the relation between development of fatigue during stress and degree of physiological recovery and thus our finding of a correlation between lack of blood pressure recovery and fatigue development during stress should be further investigated. It must be emphasized that the correlation was weak and may be a chance finding because of the large number of correlations performed. Nevertheless, the correlation may indicate that psychological mechanisms are important when considering the mechanisms for the protracted vascular response. Moreover, the correlation between the blood pressure and finger skin blood flow recovery variables may point to a common mechanism responsible for the lack of recovery in these two variables.

Steptoe (2003) proposed sustained changes in centrally mediated neurogenic vasoconstriction, or disturbance of nitric-oxide-dependent endothelial function, as explanations for lack of recovery of blood pressure after mental stress [21]. However, theories for mechanisms underlying the lack of blood pressure recovery are speculative at this stage.

## Conclusion

In the present study of healthy subjects exposed to mental stress in 60 minutes the blood pressure and acral finger skin blood flow response did not recover to baseline even after 30 minutes rest. This was in clear contrast to other physiological stress response variables (heart rate, respiration frequency and muscle activity) which recovered to baseline values early in the rest period. The protracted blood pressure response was correlated to fatigue development, but not to pain development, possibly implicating psychological mechanisms. However, because of the large number of correlations performed in the present study, one must keep in mind that this correlation may be a chance finding. The results imply that a long recovery period is necessary when the physiological recovery to mental stress is studied. Moreover, a thorough exploration of different aspects of the subjective complaints that develops during and after low-grade stress of long duration is needed. Examplewise, a valid and reliable way to distinguish between mild fatigue and unpleasantness in contrast to pain should be established in later studies of the relation between stress and development of subjective complaints. Furthermore, the duration of stress period may be of importance and should be addressed in future studies of physiological recovery after mental stress. Finally, further studies should in a prospective design investigate whether healthy subjects with a slow vascular recovery after mental stress is at risk for developing chronic stress-related disorders later in life.

## Competing interests

The authors declare that they have no competing interests.

## Authors' contributions

KBN participated in study design, in collecting the data, carried out the analysis and drafted the manuscript. TS participated in the design, advised and assisted in the statistical analysis and in the progress and drafting of the manuscript. LJS participated in the study design and in the progress of the manuscript. RBL participated in the statistical analysis and in the progress of the manuscript. RHW participated in the design, and in the progress and drafting of the manuscript. All authors read and approved the final manuscript

## Pre-publication history

The pre-publication history for this paper can be accessed here:


